# Modeling Emotions Associated With Novelty at Variable Uncertainty Levels: A Bayesian Approach

**DOI:** 10.3389/fncom.2019.00002

**Published:** 2019-01-24

**Authors:** Hideyoshi Yanagisawa, Oto Kawamata, Kazutaka Ueda

**Affiliations:** ^1^Design Engineering Laboratory, Department of Mechanical Engineering, The University of Tokyo, Tokyo, Japan; ^2^Creative Design Laboratory, Department of Mechanical Engineering, The University of Tokyo, Tokyo, Japan

**Keywords:** novelty, emotion, information, arousal, valence, uncertainty, P300, surprise

## Abstract

Acceptance of novelty depends on the receiver's emotional state. This paper proposes a novel mathematical model for predicting emotions elicited by the novelty of an event under different conditions. It models two emotion dimensions, arousal and valence, and considers different uncertainty levels. A state transition from before experiencing an event to afterwards is assumed, and a Bayesian model estimates a posterior distribution as being proportional to the product of a prior distribution and a likelihood function. Our model uses Kullback-Leibler divergence of the posterior from the prior, which we termed information gain, to represent arousal levels because it corresponds to surprise, a high-arousal emotion, upon experiencing a novel event. Based on Berlyne's hedonic function, we formalized valence as a summation of reward and aversion systems that are modeled as sigmoid functions of information gain. We derived information gain as a function of prediction errors (i.e., differences between the mean of the posterior and the peak likelihood), uncertainty (i.e., variance of the prior that is proportional to prior entropy), and noise (i.e., variance of the likelihood function). This functional model predicted an interaction effect of prediction errors and uncertainty on information gain, which we termed the arousal crossover effect. This effect means that the greater the uncertainty, the greater the information gain for a small prediction error. However, for large prediction errors, greater uncertainty means a smaller information gain. To verify this effect, we conducted an experiment with participants who watched short videos in which different percussion instruments were played. We varied uncertainty levels by using familiar and unfamiliar instruments, and we varied prediction error magnitudes by including congruent or incongruent percussive sounds in the videos. Event-related potential P300 amplitudes and subjective reports of surprise in response to the percussive sounds were used as measures of arousal levels, and the findings supported the hypothesized arousal crossover effect. The concordance between our model's predictions and our experimental results suggests that Bayesian information gain can be decomposed into uncertainty and prediction errors and is a valid measure of emotional arousal. Our model's predictions of arousal may help identify positively accepted novelty.

## Introduction

Novelty is a factor of creativity. Acceptance of novelty, however, depends on the receiver's emotions. As the “most advanced yet acceptable” (MAYA) principle of industrial designer Raymond Loewy (1951) suggested, an extremely advanced (i.e., novel) design may not be accepted. In design aesthetics, Hekkert et al. (2003) observed experimentally that both typicality and novelty affect product design preferences in ways consistent with the MAYA principle. Berlyne (1970) suggested that novelty, which he termed a collative variable, is a source of arousal potential. According to his theory, an appropriate level of arousal potential might induce a positive hedonic response, but an extreme arousal potential might induce negative responses. Several experimental studies have supported Berlyne's theory, including studies on food preferences (Giacalone et al., 2014) and artistic preferences (Silvia, 2005). However, Berlyne's model did not mathematically formalize novelty or its effects on emotions, and biases due to factors such as one's prior knowledge and experience were not exhaustively investigated. Experiments with multiple participants are required to identify the effect of novelty on the emotional response to each target and condition. The objective of this study was to mathematically model emotions elicited by novelty in order to predict how novelty affects emotions. In doing so, we aimed to provide fundamental knowledge of how to achieve acceptable novelty. Most dimensional models of emotion incorporate dimensions for arousal (or intensity) and valence (i.e., positivity or negativity) (Russell, 1980; Lang, 1995). We therefore proposed a mathematical model incorporating arousal and valence dimensions through an information theory approach. We used this model to analyze how the uncertainty of expectations prior to a novel event and the difference between expectations and reality (i.e., prediction errors) interactively affect emotional arousal. We tested our model's predictions by conducting an experiment in which participants watched short videos of percussion instruments. In the experiment, we induced uncertainty of expectations by showing instruments of varying probable familiarity, and we used inconsistencies between the instrument shown and the sound played to model prediction errors. We evaluated participants' responses to the videos by analyzing event-related potentials (ERPs) and subjective reports of feelings of surprise.

## Model of Emotional Dimensions Elicited by A Novel Event

### Overview

Novelty provides new information. We assume that the amount of information gained from an event represents the degree of novelty. The information content of an event *x* can be described as *I*(*x*) = −log*p*_*x*_, where *p*_*x*_ is the probability of *x*. *I*(*x*) is termed self-information. The self-information averaged over a probability density is termed information entropy, which Shannon et al. (1949) defined as follows:

(1) $$\begin{array}{r} H\left(X\right)=-\sum_{x\in X}p_{x}\log p_{x} \end{array}$$

For the continuous random variable *X* following a probability density distribution *p*(*x*), information entropy is expressed as:

(2) $$\begin{array}{r} H\left(X\right)=-\overset{\infty }{\underset{-\infty }{\int }}p\left(x\right)\log p\left(x\right)dx \end{array}$$

Assume a state transition from before an event to afterwards. Let the probability density distribution of a continuous random variable *x* before an event occurs, which we term the *prior*, be *q*(*x*), and let the probability density distribution of *x* after an event occurs, which we term the *posterior*, be *p*(*x*). The information entropy of the prior represents the expectation of information content gained after an event occurs or the uncertainty of prior expectations. Information content gained after an event occurs corresponds to the decrement of information entropy over the posterior. Thus, the information content gained is obtained by subtracting prior self-information from posterior self-information and averaging over the posterior:

(3) $$\begin{array}{r} {\langle -\log q\left(x\right)-\left(-\log p\left(x\right)\right)\rangle }_{p}=\overset{\infty }{\underset{-\infty }{\int }}p\left(x\right)\log\frac{p\left(x\right)}{q\left(x\right)}dx\equiv D_{KL}\left(p\left(x\right)||q\left(x\right)\right) \end{array}$$

where < *q*>_*p*_ represents the average of density *q* over density *p*. The expression *D*_*KL*_(*p*||*q*) is the Kullback-Leibler (KL) divergence of *p* from *q* (Kullback and Leibler, 1951). Hereinafter, we term the KL divergence of the Bayesian posterior from the prior the *information gain*. The more novel an event is, the more information one gains. Information gain represents averaged surprise. Itti and Baldi (2009) defined the KL divergence of the Bayesian posterior from the prior as surprise and provided experimental evidence that it attracts visual attention.

Surprise is often used as a typical high-arousal emotion (Mauss and Robinson, 2009). Thus, we used the information gain as a mathematical expression of the arousal dimension of emotion. We then investigated the valence dimensions. An event with no information causes no arousal and has a neutral valence. Conversely, excessive information gain, such that one can hardly cope, should cause discomfort (i.e., a negative valence). Therefore, we hypothesized that one positively accepts a novel event providing an appropriate amount of information gain that can be coped with. Based on the arousal potential model (Berlyne, 1970), we formulated the valence as a function of information gain.

### Bayesian Model

Bayes's theorem provides a formula for updating the prior to the posterior. Recent studies have indicated that humans perform near-optimal Bayesian inference (Ma et al., 2006) in a wide variety of tasks, ranging from cue integration (Ernst and Banks, 2002; Kersten et al., 2004; Stocker and Simoncelli, 2006; Yanagisawa, 2016) to decision-making and motor control (Körding and Wolpert, 2004, 2006). Let a prior be π(θ) in terms of a parameter *θ* that one estimates. After one obtains continuous data *x* ∈ *R* by experiencing an event, the prior π(*θ*) is updated to the posterior π(*θ*|*x*) according to the following formula derived from Bayes's theorem:

(4) $$\begin{array}{r} \pi \left(\theta |x\right)=\frac{f\left(x|\theta \right)\pi \left(\theta \right)}{\underset{\theta }{\int }f\left(x|\theta \right)\pi \left(\theta \right)d\theta }\propto f\left(x|\theta \right)\pi \left(\theta \right) \end{array}$$

where *f*(*x*|θ) is the likelihood function of θ when data *x* are obtained. The posterior is proportional to the product of the likelihood function and the prior.

**Figure 1:**
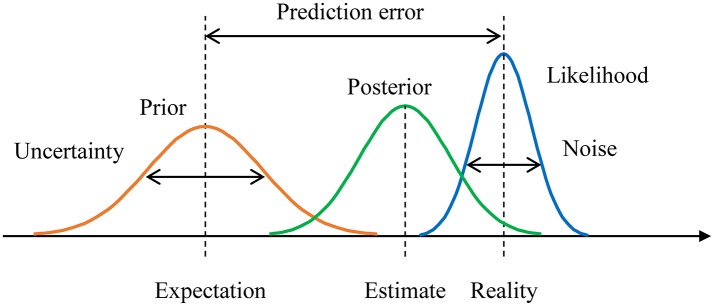
Example of Bayesian inference with a prior distribution, a posterior distribution, and a likelihood function. The prediction error is the difference between the prior expectation and the peak of the likelihood function (i.e., reality). Uncertainty is the variance of the prior. Noise is the variance of the likelihood function.

Figure 1 shows an example of the relationships between the prior, the posterior, and the likelihood function. Neural population activity with Poisson variability can encode any Gaussian probability distribution (Ma et al., 2006). With Poisson variability, the posterior with a flat prior converges to a Gaussian distribution as the number of neurons increases. The mean of the Gaussian distribution is close to the stimulus at which the population activity peaks. The variance of the distribution is encoded as a value that is inversely proportional to the gain of the population code (i.e., the distribution's amplitude). Hence, we assume Gaussian distributions for the prior and the likelihood function. Assume one obtains *n* samples of event *x* and encodes them as a Gaussian posterior *N*(μ, σ^2^) with a flat prior. Now assume a non-flat prior of μ that follows a Gaussian distribution *N*(η, τ^2^). Using Bayes's theorem, the prior is updated to a Gaussian distribution $N\left(\eta_{post},{\tau_{post}}^{2}\right)$, where:

(5) $$\begin{array}{r} Average:\eta_{post}=\frac{s_{p}\bar{x}+s_{l}\eta }{s_{p}+s_{l}};Variance:{\sigma_{post}}^{2}=\frac{s_{p}s_{l}}{s_{p}+s_{l}} \end{array}$$

In these formulae, $\bar{x}$ is the mean of the data, $s_{p}=\tau^{2}$, and $s_{l}=\sigma^{2}/n$. Therefore, the prior and the posterior are represented as the following Gaussian functions, respectively:

(6) $$\begin{array}{rcl} \pi \left(\mu \right) & = & N\left(\mu ;\eta ,s_{p}\right)=\frac{1}{\sqrt{2\pi s_{p}}}\exp\left[-\frac{{\left(\mu -\eta \right)}^{2}}{2s_{p}}\right],and \end{array}$$

(7) $$\begin{array}{rcl} \pi \left(\mu |x\right) & = & N\left(\mu ;\eta_{post},{\sigma_{post}}^{2}\right)=\frac{1}{\sqrt{2\pi {\sigma_{post}}^{2}}}\exp\left[-\frac{{\left(\mu -\eta_{post}\right)}^{2}}{2{\sigma_{post}}^{2}}\right] \end{array}$$

### A Functional Model of Emotional Arousal

As noted in Overview, we represented emotional arousal as information gain after experiencing an event. The information gain from the prior to the posterior *D*_*KL*_(π(μ|*x*)||π(μ))≡*G* can be derived from formulae (2, 5, 6, and 7) as the following formula:

(8) $$\begin{array}{rcl} G & = & \overset{\infty }{\underset{-\infty }{\int }}\pi \left(\mu |x\right)\log\frac{\pi \left(\mu |x\right)}{\pi \left(\mu \right)}d\mu \end{array}$$

$$\begin{array}{rc} = & \frac{1}{2}\left\{\frac{s_{p}}{{\left(s_{p}+s_{l}\right)}^{2}}\delta^{2}+\log\frac{s_{p}+s_{l}}{s_{l}}-\frac{s_{p}}{s_{p}+s_{l}}\right\} \end{array}$$

where δ is the difference between the prior expectation (η) and the peak of the likelihood function ($\bar{x}$). δ represents the difference between expectations and reality, so we term δ the *prediction error* (Yanagisawa, 2016) (Figure 1).

Information entropy of the prior is proportional to a logarithm of τ as follows:

(9) $$\begin{array}{r} H_{prior}=-\overset{\infty }{\underset{-\infty }{\int }}\pi \left(\mu \right)\log\pi \left(\mu \right)d\mu =\log\sqrt{2\pi e}\log\tau \propto \log\tau \end{array}$$

Thus, we term *s*_*p*_ the *uncertainty* (Yanagisawa, 2016), and *s*_*l*_ represents the variation of data *x*. In the case of sensory data (i.e., stimuli), the variance refers to *external noise* (Yanagisawa, 2016). From formula (7), we can regard the information gain *G* as a function of the prediction error δ , the uncertainty *s*_*p*_, and the external noise *s*_*l*_:

(10) $$\begin{array}{r} G=f\left(\delta ,s_{p},s_{l}\right) \end{array}$$

### Interaction Effect of Uncertainty and Prediction Errors on Information Gain

We analyzed how prediction errors, uncertainty, and external noise affect information gain (i.e., arousal levels). In formula (8), information gain is a quadratic function of the prediction error δ when uncertainty and external noise are fixed.

(11) $$\begin{array}{rcl} G & = & \alpha \delta^{2}+\beta , \end{array}$$

$$\begin{array}{rcl} \alpha & = & \frac{s_{p}}{2{\left(s_{p}+s_{l}\right)}^{2}},and\\ \beta & = & \frac{1}{2}\left(log\frac{s_{p}+s_{l}}{s_{l}}-\frac{s_{p}}{s_{p}+s_{l}}\right) \end{array}$$

The value of α is always greater than zero because *s*_*p*_ and *s*_*l*_ are variances that are always greater than zero. Thus, the information gain is a monotonically increasing function of a prediction error. This means that the level of an arousal dimension, such as the degree of surprise, is proportional to the square of the difference between expectations and reality.

Next, we investigated the effect of uncertainty. We found that the partial derivative of the intercept β with respect to uncertainty *s*_*p*_ is always less than zero:

(12) $$\begin{array}{r} \frac{\partial \beta }{\partial s_{p}}=\frac{s_{p}}{{2(s_{p}+s_{l})}^{2}}>0 \end{array}$$

Thus, at δ = 0, the greater the uncertainty, the greater the information gain. We then investigated the case of δ > 0. We compared any two information gain functions of δ using formula (10) with constant external noise between different degrees of uncertainty. If the two functions of different uncertainties have an intersection, then the information gains change as δ increases. We then assumed two information gain functions with different uncertainties, *G*_1_ and *G*_2_:

$$\begin{array}{rcl} G_{1} & = & \alpha_{1}\delta^{2}+\beta_{1}\;and\; \end{array}$$

(13) $$\begin{array}{rcl} G_{2} & = & \alpha_{2}\delta^{2}+\beta_{2} \end{array}$$

A condition where the two functions have an intersection is α_1_δ^2^ + β_1_ = α_2_δ^2^ + β_2_. We derived δ^2^ (α_1_ − α_2_) + (β_1_ − β_2_) = 0 under β_1_ ≠ β_2_. Therefore, (α_1_ − α_2_)/(β_1_ − β_2_) < 0 is the condition. We found that this condition applies when the relationship between different uncertainties *s*_*p*1_ and *s*_*p*2_ and constant external noise *s*_*l*_ is as follows:

(14) $$\begin{array}{r} s_{p1}s_{p2}>{s_{l}}^{2} \end{array}$$

Because the uncertainty of prediction is likely to exceed the external noise (i.e., the uncertainty of sensory stimuli), the condition in question is likely to occur. Given formula (12), the greater the uncertainty, the greater the intercept of the information gain function. As the prediction error increases, the difference in information gains between the two functions changes such that lower uncertainty tends to mean greater information gain.

**Figure 2:**
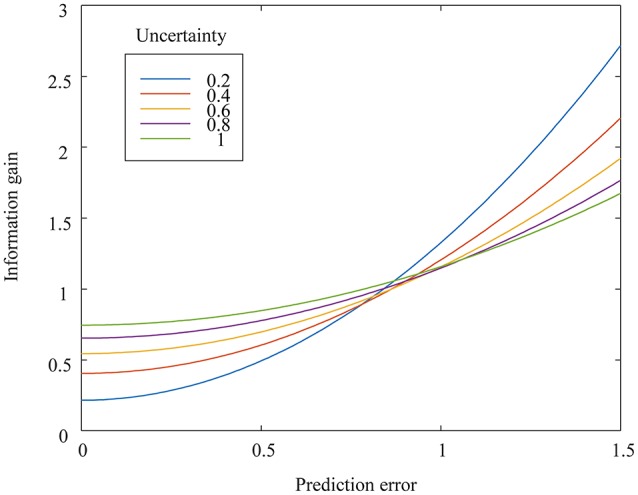
Mathematically derived information gain, as a function of prediction errors, for uncertainty levels varying from 0.2 to 1.0. The external noise is set at 0.1.

Figure 2 shows two functions of information gain with respect to different uncertainties at constant external noise. The two information gain functions have an intersection point. The information gain as an index of arousal (in this case, surprise) increases as the prediction error increases. The prediction error and uncertainty have an interaction effect on information gain. The greater the uncertainty, the greater the information gain for zero or small prediction errors. The smaller the uncertainty, the greater the information gain for larger prediction errors. We term this intersection-related phenomenon the *arousal crossover effect*.

### A Functional Model of Emotional Valence

**Figure 3:**
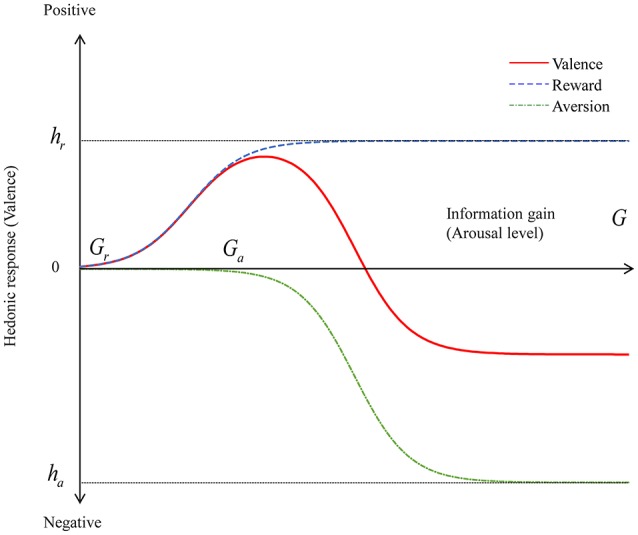
Valence as a function of information gain. The valence is modeled as a summation of two sigmoidal functions representing reward and aversion systems.

We next investigated how novelty affects the valence dimensions of positivity and negativity. Berlyne (1970) proposed collative variables that consist of stimulus factors, such as novelty, complexity, uncertainty, and conflict. Each collative variable has the quality of arousal potential (i.e., the ability to affect the intensity of arousal). Highly novel stimuli can increase arousal. Berlyne (1967, 1971) assumed that the hedonic qualities of stimuli arise from separate biological incentivization systems. The first system, the *reward system*, generates positive affect whenever arousal potential increases. The second system, the *aversion system*, generates negative affect whenever arousal potential increases. The aversion system has a higher absolute activation threshold than the reward system does. Thus, the joint operation of these two systems creates an inverted U-shaped curve, as shown in Figure 3. The valence of a stimulus changes from neutral to positive as the arousal potential increases but shifts from positive to negative after the arousal potential passes the peak positive valence. This inverse U shape is reasonable. One may feel safe and experience boredom if stimuli are too familiar (i.e., not novel). Conversely, one may feel uncomfortable if stimuli are extremely unfamiliar and novel. However, in Bayesian models, repeated exposure to the same stimulus decreases both prediction errors and uncertainty. Thus, the iterative information gain for each update decreases. The decreasing information gain and the inverse U-shaped function may explain emotional desensitization, which is the psychological phenomenon of emotional responsiveness to a negative, aversive, or positive stimulus diminishing after repeated exposure to it. The positive hedonic response to a stimulus is diminished by decreasing information gain after repeated exposure to it, and a negative hedonic response to an extremely novel stimulus is shifted to a positive or neutral response by decreasing information gain after repeated exposure.

As noted in Overview, we formalized the arousal level as information gain from an event. If an event does not provide any information, then the valence can be neutral. At the opposite extreme, if an event provides excessive information that is difficult for the brain to process, then the valence can become negative. We can reasonably assume that between these two extremes there lies a “sweet spot” at which an optimum information gain maximizes a positive valence. We formalized valence as a summation of the reward and aversion systems and used sigmoid functions (Saunders, 2012) to model information gain for each system:

(15) $$\begin{array}{rcl} Valence & = & Reward+Aversion \end{array}$$

(16) $$\begin{array}{rcl} where\;Reward\left(G\right) & = & \frac{h_{r}}{1+\exp\left(-c_{r}G+G_{r}\right)} \end{array}$$

$$\begin{array}{rcl} and\;Aversion\left(G\right) & = & \frac{-h_{a}}{1+\exp\left(-c_{a}G+G_{a}\right)} \end{array}$$

In these formulae, *G*_*r*_ and *G*_*a*_ represent the thresholds of information gain that activate reward and aversion systems, respectively. The variables *h*_*r*_ and *h*_*a*_ are the maxima of positive and negative valence levels, respectively, and *c*_*r*_ and *c*_*a*_ represent the respective gradients. The condition *G*_*r*_<*G*_*a*_ must always be satisfied because the threshold of the reward system is lower than that of the aversion system. If an extreme information gain occurs, then the condition *h*_*r*_<*h*_*a*_ must be satisfied to obtain a negative valence. Figure 3 shows the valence, reward, and aversion functions of formula (15). We can observe that the valence function is an inverse U-shaped curve.

### Model Summary

**Figure 4:**
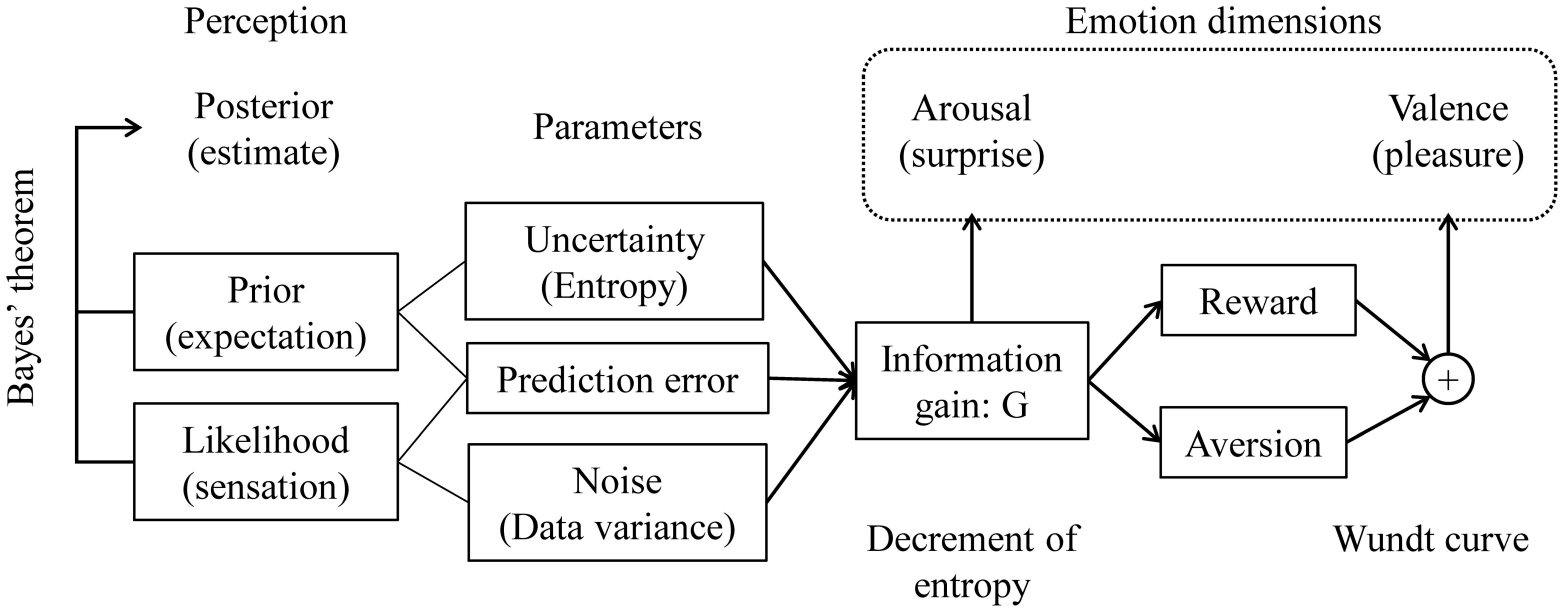
Proposed model of the dimensions of novelty-elicited emotions. Emotional arousal is expressed as information gain from the Bayesian prior to the posterior. Valence is a summation of reward and aversion systems, which are functions of information gain.

Figure 4 shows a schematic of our proposed model. We formalized emotional arousal using information gain from an event, which we represented as the KL divergence from the prior to the posterior. We derived the information gain as a function of three parameters: uncertainty, the prediction error, and external noise, which are represented as the variance of the prior (or entropy), the difference between the prior expectation and the peak of the likelihood function, and the variance of the likelihood function, respectively. We formulated valence (i.e., positivity or negativity) as a summation of reward and aversion systems represented as information gain functions based on Berlyne's theory.

In our model, the information gain is a key parameter to explain the emotional dimensions of arousal and valence. The information gain increases as the prediction error increases. Recent neurological studies have shown that dopaminergic neurons encode the prediction error signal of reward (Schultz et al., 1997; Bayer and Glimcher, 2005). Our model explains a reward system as a function of information gain affected by prediction errors. From a mathematical analysis, we found that uncertainty and prediction errors have interaction effects on information gain. Prediction errors increase information gain. The greater the uncertainty, the more the information gain for zero or small prediction errors. In contrast, the smaller the uncertainty, the more the information gain for large prediction errors. Uncertainty represents the degree of belief in the prior expectation. The familiarity of an event or target and one's knowledge and experience of a target affect uncertainty. For example, if a product is so familiar that everyone knows it well, then uncertainty about the product is small. In contrast, if a product is unfamiliar, then uncertainty about the product should be considerable. Thus, uncertainty represents prior information before experiencing a target event. Indeed, uncertainty is proportional to the information entropy of the prior, as in formula (9). This model suggests that emotion is influenced by prior information, discrepancies between expectations and reality, and stimulus attributes.

## Effects of Uncertainty and Prediction Errors on Emotional Arousal Related to Percussion Instruments

We investigated the effects of uncertainty and prediction errors on surprise to validate the arousal crossover effect derived from the mathematical model in Interaction Effect of Uncertainty and Prediction Errors on Information Gain. Specifically, we tested the hypothesis that uncertainty increases surprise when prediction errors are small and decreases surprise when prediction errors are large. A set of short videos featuring percussion instruments and accompanying sounds were used as stimuli. In each video, a percussion instrument was presented and then beaten. Different percussive sounds were synthesized. We assumed a transition from a visual prior (i.e., the appearance of an instrument) to an auditory posterior (i.e., the percussive sound). Participants predicted an instrument's sound from its appearance and then listened to a sound. We induced prediction errors by manipulating the congruency between the synthesized percussive sounds and the instrument shown. We assumed that prediction errors were large when the synthesized percussive sounds were incongruent with the instruments shown, and we assumed that the familiarity or unfamiliarity of the instruments shown produced different levels of uncertainty. The appearance of a familiar percussion instrument, such as a hand drum, produces certainty of expectations concerning its sound (i.e., a small uncertainty). The appearance of an unfamiliar percussion instrument, such as the African percussion instrument known as the jawbone, produces uncertain expectations concerning its sound (i.e., a large uncertainty).

We used both questionnaires and ERP recordings to assess participants' levels of surprise in response to the percussive sound in each video. We quantified surprise intensities based on responses to a four-level Likert scale and measurements of ERP P300 amplitudes (Mars et al., 2008).

### Methods

#### Participants

Nine right-handed healthy male volunteers (mean age ± standard deviation: 21.7 ± 1.2 years; range: 20–24 years) with normal or corrected-to-normal vision and hearing participated in this study. The study protocol was approved by the Ethics Committee of the Graduate School of Engineering at the University of Tokyo. In accordance with the principles of the Declaration of Helsinki, all participants provided written informed consent prior to their participation in this study. The participants were allowed to interrupt the experiment sessions at their convenience.

#### Stimuli

The stimuli consisted of eight short videos in which a percussion instrument was beaten once and a synthesized percussive sound followed. Table 1 shows the combinations of instruments shown and the synthesized sounds (Videos are available in Supplementary Material). The clave and hand drum were selected as familiar percussion instruments (type A), and the jawbone and slit drum were selected as unfamiliar percussion instruments (type B). To create incongruent conditions, we synthesized percussive sounds that were inconsistent with the instruments shown. Our stimuli included videos with visually familiar instruments and congruent sounds (type AX), videos with visually familiar instruments and incongruent sounds (type AY), videos with visually unfamiliar instruments and congruent sounds (type BX), and videos with visually unfamiliar instruments and incongruent sounds (type BY).

**Table 1:** Combinations of percussion instruments and percussive sounds. (Video stimuli are available in the Supplementary Material).

	**Instrument**	**Congruent sound (X)**	**Incongruent sound (Y)**
Familiar (A)	Clave	Clave (AX), (Video S1)	Bell (AY), (Video S3)
	Hand drum	Hand drum (AX), (Video S2)	Guiro (AY), (Video S4)
Unfamiliar (B)	Jawbone	Jawbone (BX), (Video S5)	Vibraphone (BY), (Video S7)
	Slit drum	Slit drum (BX), (Video S6)	Snare (BY), (Video S8)

The duration of each video was 2,500 ms. First, a percussion instrument appeared in the center of the screen. The percussion instrument was then beaten once 500 ms into the video while a percussive sound was presented simultaneously. Each video had an 18° horizontal visual angle and a 10° vertical visual angle and was centrally presented against a black background on a 29.8-inch display located 100 cm away from the participant. The participants wore noise-canceling headphones covered by earmuffs while watching the videos.

#### Procedure

The participants completed experiments individually in an electromagnetically shielded dark room. After participants received instructions for the procedure, they were asked to start the experiment.

First, we conducted sound-only experiments in which we attempted to ensure uniform surprise levels in response to the percussive sounds used in each video type (i.e., AX, AY, BX, and BY). Achieving this uniformity was necessary so that we could be sure that our observations in later experiments with audiovisual stimuli reflected the effects of visual priors. The eight percussive sounds were presented to the participants via headphones in five random-order sets without any visual stimuli. This phase of the procedure consisted of 40 trials (eight sounds × five presentation sets). The interstimulus interval (ISI) was 1,000–2,000 ms, with an average of 1,500 ms.

Second, we conducted additional sound-only experiments in which we used electroencephalography (EEG) to confirm the uniformity of the surprise levels evoked by the percussive sounds of each video type. The eight percussive sounds were presented to the participants via headphones in 20 random-order sets without any visual stimuli. This phase of the procedure consisted of 160 trials (eight sounds × 20 presentation sets). The ISI was 1,000–2,000 ms, with an average of 1,500 ms. EEG recordings were obtained for each trial. A short break was inserted after the tenth presentation set.

Third, the participants watched videos of a clave or a hand drum, which we assumed were familiar instruments for our participants, accompanied by congruent percussive sounds. The videos thus belonged to type AX. The participants watched these videos five times to create expectations of certainty and congruity.

Lastly, we conducted the main experiment in which participants watched videos while undergoing EEG recordings and subjectively reporting feelings of surprise. The eight videos described in Table 1 were presented to the participants in 20 random-order sets. This phase of the procedure consisted of 160 trials (eight videos × 20 presentation sets). The ISI was 1,000–2,000 ms, with an average of 1,500 ms. EEG recordings were obtained for each trial. A short break was inserted after the tenth presentation set. During the first, tenth, and final presentation sets, the participants used a four-level Likert scale to report the intensities of their surprise upon listening to the percussive sounds. The participants used four push buttons under their fingers to provide these reports so that they did not have to avert their eyes from the display.

#### EEG Recordings

The EEG data were recorded with a portable digital recorder (Polymate AP1132, TEAC Corporation, Tokyo, Japan) and active electrodes. The data were obtained from three midline electrodes positioned at the Fz, Cz, and Pz points as defined by the international 10–20 system with reference to the nose. The data were recorded at a sampling rate of 500 Hz. The time constant was set at 3 s. All electrode impedances were below 50 kΩ. A digital bandpass filter of 0.1–20 Hz was applied.

#### EEG Data Analysis

The ERP waveforms were obtained by averaging data from the period starting 200 ms before the stimulus onset, which we define as the start of the video in video stimulus sessions, and ending 1,500 ms after the stimulus onset. This averaging was done separately for each participant, stimulus type (i.e., AX, AY, BX, and BY), and electrode site for both the sound-only and video stimuli. For each averaged waveform, the 200-ms period preceding the stimulus onset was defined as the baseline. Any epochs containing EEG signals exceeding ± 100 μV were regarded as eye movement–related artifacts and automatically removed. The P300 component was designated as the largest positive peak occurring 250–600 ms after the onset of the percussive sound. The baseline-to-peak P300 amplitudes were measured at the Pz point, which was the dominant electrode site.

#### Statistical Analysis

Repeated-measures analysis of variance (ANOVA) was applied to the ERP and Likert scale data. One-way ANOVA of the P300 data from the sound-only sessions was conducted to examine how different percussive sound types affected P300 amplitudes. To identify interaction effects on surprise intensities, we analyzed the P300 amplitude and Likert scale data from the video sessions with two-way ANOVA in terms of congruity and familiarity. Statistical significance was defined as *p* < 0.05 for all statistical tests. We compared the experimental results to the simulation results shown in Figure 2.

### Experimental Results

The type of percussive sound did not significantly affect P300 amplitudes in the sound-only sessions (*F* = 0.35, *p* = 0.79).

**Figure 5:**
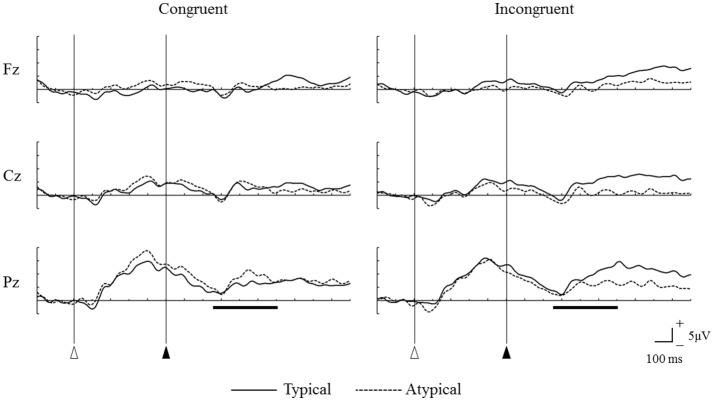
Grand mean event-related potential waveforms for the four different video types measured from frontal (Fz), central (Cz), and parietal (Pz) midline regions. Open triangles represent the onsets of the videos, and solid triangle represent the onsets of percussive sounds. The horizontal bars show the time range of 250–600 ms after the onset of the percussive sound.

Figure 5 shows the grand mean ERP waveforms for the four video types in the main video session. Under the congruent condition, the sounds of unfamiliar percussion instruments (type BX) elicited larger P300 amplitudes than the sounds of familiar percussion instruments (type AX) did. However, under the incongruent condition, the sounds of familiar percussion instruments (type AY) elicited larger P300 amplitudes than the sounds of unfamiliar percussion instruments (type BY) did.

**Figure 6:**
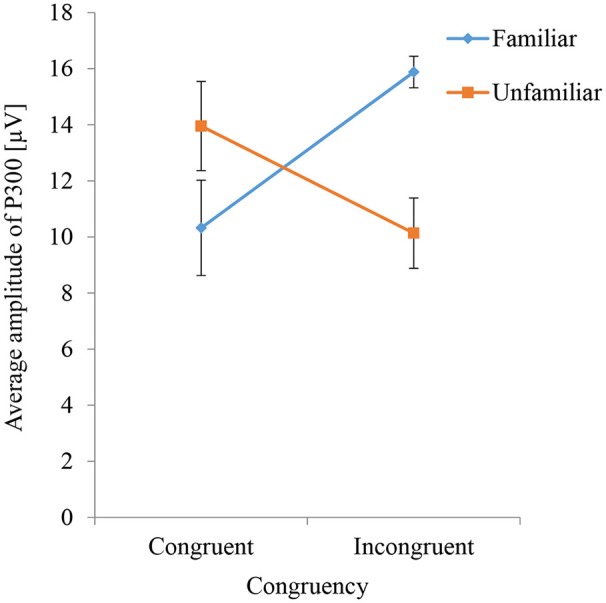
P300 amplitudes evoked by percussive sounds that are congruent or incongruent with the instrument shown. The results for familiar and unfamiliar instruments are compared.

Figure 6 shows the averaged P300 amplitude for each condition (i.e., all combinations of congruity and familiarity) in the main video session. The interaction effect of congruity and familiarity on P300 amplitudes was significant (*F* = 10.99, *p* = 0.01). The simple main effect of familiarity was significant for both congruent (*F* = 4.7, *p* = 0.047) and incongruent (*F* = 11.82, *p* = 0.004) sounds. When congruent sounds were played, the average P300 amplitude for the unfamiliar instruments was larger than that for the familiar instruments, but when incongruent sounds were played, the average P300 amplitude for the unfamiliar instruments was smaller than that for the familiar instruments. The simple main effect of congruity was significant for the familiar instruments (*F* = 6.5, *p* = 0.02) but not for the unfamiliar instruments (*F* = 3.09, *p* = 0.09). Thus, the average P300 amplitude evoked by incongruent sounds was larger than that evoked by congruent sounds only when familiar instruments were shown.

**Figure 7:**
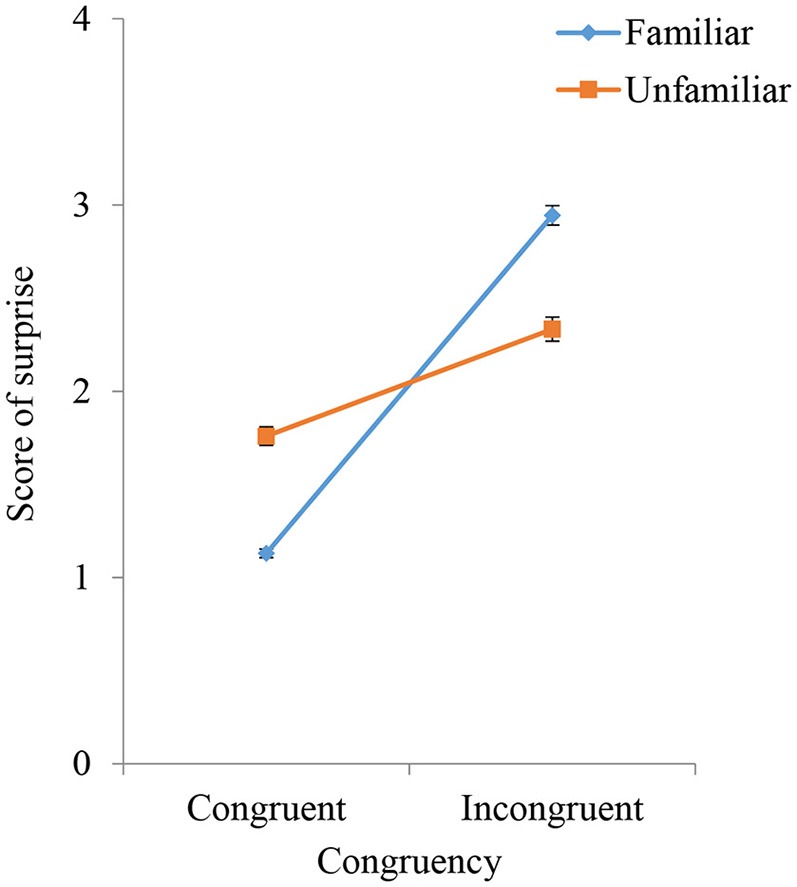
Subjectively reported scores for surprise intensities in response to percussive sounds that are congruent or incongruent with the instrument shown. The results for familiar and unfamiliar instruments are compared.

Figure 7 shows the average Likert scale surprise rating for each stimulus used in the main video session under different conditions of congruity and familiarity. The interaction effect of congruity and familiarity was significant (*F* = 39.06, *p* < 0.001), as was the simple main effect of congruity (*F* = 144.9, *p* < 0.001). The simple main effect of familiarity was significant for both congruent (*F* = 167.14, *p* < 0.001) and incongruent (*F* = 16.72, *p* < 0.001) sounds. The difference between Likert scale surprise ratings for the familiar and unfamiliar instruments was significant under both congruent and incongruent sound conditions (*p* < 0.01). These results show that subjectively rated surprise under the unfamiliar instrument condition was greater than that under the familiar instrument condition when the sounds were congruent but that subjectively rated surprise under the familiar instrument condition was greater than that under the unfamiliar instrument condition when the sounds were incongruent. The crossover in both Figures 6, 7 corresponds to the simulation result in Figure 2.

## Discussion

We assumed that information gain from an event, which can be calculated using KL divergence between the Bayesian prior and the posterior, represents the intensity of arousal emotions such as surprise. Prediction errors, which are differences between prior expectations and likelihood function peaks, increase information gain and surprise. We conducted an experiment featuring videos of percussion instruments accompanied by synthesized percussive sounds. We varied uncertainty levels by using familiar and unfamiliar instruments, and we varied prediction error magnitudes by using congruent or incongruent percussive sounds. We used ERP P300 amplitudes and subjective reports to assess the participants' surprise levels in response to the percussive sounds. Compared to congruent sounds, incongruent sounds produced greater subjectively reported surprise intensities, and this was particularly true when familiar percussion instruments were shown. Similarly, incongruent sounds increased P300 amplitudes when familiar percussion instruments were shown. These results suggest that prediction errors related to visuoauditory incongruities increase surprise, attention, and the amount of information processed in the brain (i.e., the arousal level). Moreover, instrument familiarity, which induces certainty of expectations concerning percussive sounds, provides a greater potential for arousal in the event of visuoauditory incongruity than is possible with unfamiliar instruments, which induce uncertainty of expectations concerning sounds. This result supports our mathematical hypothesis that information gain serves as an index of arousal.

We mathematically derived a hypothesized effect that we termed the *arousal crossover effect*: uncertainty, represented as variance of the prior, increases information gain when prediction errors are zero or small, but uncertainty decreases information gain when prediction errors are large. Both the P300 amplitude data and the subjectively reported surprise intensity data supported this hypothesized effect. When congruent sounds accompanied the instruments shown, videos featuring unfamiliar instruments evoked greater P300 amplitudes and subjectively reported surprise scores than videos featuring familiar instruments did. However, when incongruent sounds accompanied the instruments shown, videos featuring unfamiliar instruments evoked lower P300 amplitudes and subjectively reported surprise scores than videos featuring familiar instruments did.

This concordance between our proposed model's predictions and the experimental results suggests that information gain obtained from a novel event represents the level of emotional arousal. Previous studies have shown that the KL divergence represents surprise that attracts human attention (Itti and Baldi, 2009). We newly formalized the information gain, which is mathematically equivalent to KL divergence, as a function of prediction errors, uncertainty, and noise and showed both mathematically and experimentally that an interaction effect of prediction errors and uncertainty exists. Uncertainty of the prior depends on an individual's knowledge and prior experiences as well as the familiarity of an event. Prior knowledge and experience produce certainty of expectations. This implies that our proposed model may explain individual differences in emotional responses to an identical novel event as resulting from differences in knowledge and prior experience. For example, an expert's expectations should be more certain than those of a novice. Using our model, we can therefore predict that novices are more surprised than experts are when an event differs marginally from prior expectations but that experts are more surprised than novices are when an event greatly differs from prior expectations.

We formalized emotional valence as a function of arousal levels based on Berlyne's theory (Berlyne, 1970). The functional model forms an inverse U-shaped curve that has a positive valence peak at a certain arousal level. Therefore, we can predict that variable uncertainty levels related to an individual's knowledge and experience and the familiarity of an event modulate the effect of prediction errors on valence responses. Although our mathematical model is firmly grounded in Berlyne's theory, further experimental evidence validating the ability of our valence model to predict empirical observations will be more than welcomed. Indeed, the chief limitation of this study is the reliance on mathematical formulations of both arousal and valence and the lack of experimental validation of our formulation of valence. In future studies, we will conduct experiments to test the validity of our valence model.

## Author Contributions

HY designed and supervised the study and formalized the mathematical model. HY, OK, and KU designed and conducted the experiment. OK and KU measured and analyzed the EEG data. HY and KU drafted the manuscript. All authors revised the manuscript.

### Conflict of Interest Statement

The authors declare that the research was conducted in the absence of any commercial or financial relationships that could be construed as a potential conflict of interest.

## Supplementary Material

Video S1 Clave (AX).

Video S2 Hand drum (AX).

Video S3 Clave - Bell (AY).

Video S4 Hand drum - Guiro (AY).

Video S5 Jawbone (BX).

Video S6 Slit drum (BX).

Video S7 Jawbone - Vibraphone (BY).

Video S8 Slit drum - Snare (BY).
