# Corrigendum: Modeling Emotions Associated With Novelty at Variable Uncertainty Levels: A Bayesian Approach

**DOI:** 10.3389/fncom.2020.00027

**Published:** 2020-04-15

**Authors:** Hideyoshi Yanagisawa, Oto Kawamata, Kazutaka Ueda

**Affiliations:** ^1^Design Engineering Laboratory, Department of Mechanical Engineering, The University of Tokyo, Tokyo, Japan; ^2^Creative Design Laboratory, Department of Mechanical Engineering, The University of Tokyo, Tokyo, Japan

**Keywords:** novelty, emotion, information, arousal, valence, uncertainty, P300, surprise

In the original article, there were errors in Equations (5) and (12) and the text.

In Equation (5) there was a typographical error in the numerator and we wrote

$$\begin{array}{r} \eta_{post}=\frac{s_{p}\eta +s_{l}\bar{x}}{s_{p}+s_{l}} \end{array}$$

when it should be

$$\begin{array}{r} \eta_{post}=\frac{s_{p}\bar{x}+s_{l}\eta }{s_{p}+s_{l}} \end{array}$$

In Equation (12) it was written that uncertainty is always less than zero:

$$\begin{array}{r} \frac{\partial \beta }{\partial s_{p}}=\frac{1}{2}\left\{-\frac{s_{p}}{s_{l}\left(s_{p}+s_{l}\right)}-\frac{s_{l}}{{\left(s_{p}+s_{l}\right)}^{2}}\right\}\leq 0 \end{array}$$

This has been corrected to show that uncertainty *s*_*p*_ is always more than zero:

$$\begin{array}{r} \frac{\partial \beta }{\partial s_{p}}=\frac{s_{p}}{2{\left(s_{p}+s_{l}\right)}^{2}}>0 \end{array}$$

Similarly, there was a miscalculation in the text preceding Equation (14). We wrote “We derived $\delta^{2}+\left(\alpha_{1}-\alpha_{2}\right)/\left(\beta_{1}+\beta_{2}\right)=0$ under β_1_ ≠ β_2_. Therefore, (α_1_ − α_2_)/(β_1_ + β_2_) < 0 it is the condition.” We actually derived “$\delta^{2}\left(\alpha_{1}-\alpha_{2}\right)+\left(\beta_{1}-\beta_{2}\right)=0$ under β_1_ ≠ β_2_” and the condition is “(α_1_ − α_2_)(β_1_ − β_2_) < 0.”

A correction has been made to the Model of Emotional Dimensions Elicited by A Novel Event section, subsection Interaction Effect of Uncertainty and Prediction Errors on Information Gain, paragraph 5.

“A condition where the two functions have an intersection is $\alpha_{1}\delta^{2}+\beta_{1}$ = $\alpha_{2}\delta^{2}+\beta_{2}$. We derived $\delta^{2}\left(\alpha_{1}-\alpha_{2}\right)+\left(\beta_{1}-\beta_{2}\right)=0$ under β_1_ ≠ β_2_. Therefore, (α_1_ − α_2_)(β_1_ − β_2_) < 0 is the condition. We found that this condition applies when the relationship between different uncertainties *s*_*p*__1_ and *s*_*p*__2_ and constant external noise *s*_*l*_ is as follows:

$$\begin{array}{r} {s_{p}}_{1}{s_{p}}_{2}>{s_{l}}^{2} \end{array}$$

The authors apologize for these errors, which does not change the scientific conclusions of the article in any way. The original article has been updated.

